# Extensive Bilateral Intracranial Calcifications: A Case of Iatrogenic Hypoparathyroidism

**DOI:** 10.1155/2013/932184

**Published:** 2013-02-24

**Authors:** Vaso Zisimopoulou, Anna Siatouni, Grigorios Tsoukalos, Antonios Tavernarakis, Stylianos Gatzonis

**Affiliations:** ^1^Department of Neurology, Evaggelismos Hospital, 45-47 Ipsilantou Street, 10676 Athens, Greece; ^2^Department of Neurosurgery, Athens Medical School, Evaggelismos Hospital, 45-47 Ipsilantou Street, 10676 Athens, Greece; ^3^Department of Radiology, Evaggelismos Hospital, 45-47 Ipsilantou Street, 10676 Athens, Greece

## Abstract

This is a case of a 69-year-old male patient with long-standing iatrogenic hypoparathyroidism after total thyroidectomy. The clinical evaluation revealed mild neurological symptoms and excessive brain calcinosis. Intracranial calcification that affects structures other than the basal ganglia and the cerebellum is a rare manifestation of postoperative hypoparathyroidism. Detection of brain calcinosis in patients who had total thyroidectomy can motivate clinicians in further investigation of possible hypoparathyroidism with measurement of calcium and phosphorus serum levels.

## 1. Introduction

Intracranial calcification that affects structures other than the basal ganglia and the cerebellum is a rare manifestation of postoperative hypoparathyroidism. The incidence of hypoparathyroidism following total thyroidectomy is reported in several series between 0.3% and 6.3% for permanent hypoparathyroidism and between 5% and 22% for transient hypoparathyroidism [1]. We present a case of long-standing iatrogenic hypoparathyroidism with bilateral extensive intracranial calcifications and only mild clinical symptoms.

## 2. Case Report

**Figure 1:**
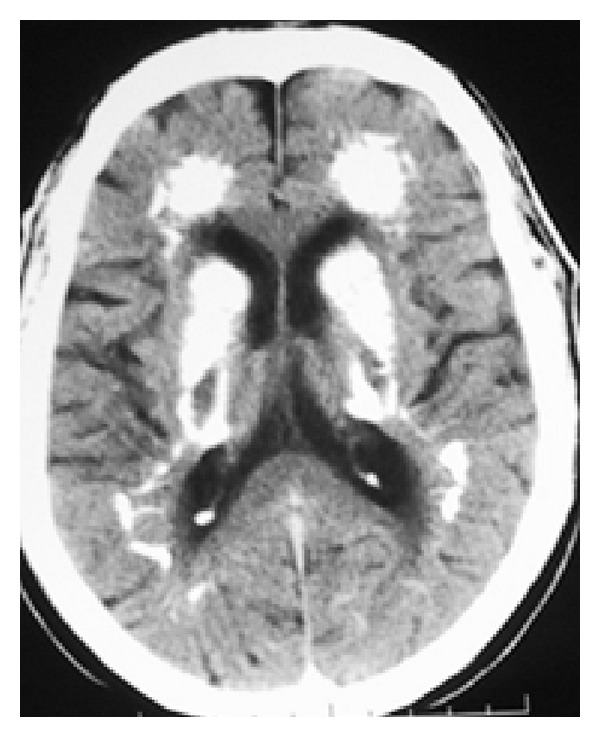
CT scan axial view demonstrating extensive bilateral calcifications in the periventricular white matter (frontal horns, basal ganglia, and internal capsule).

**Figure 2:**
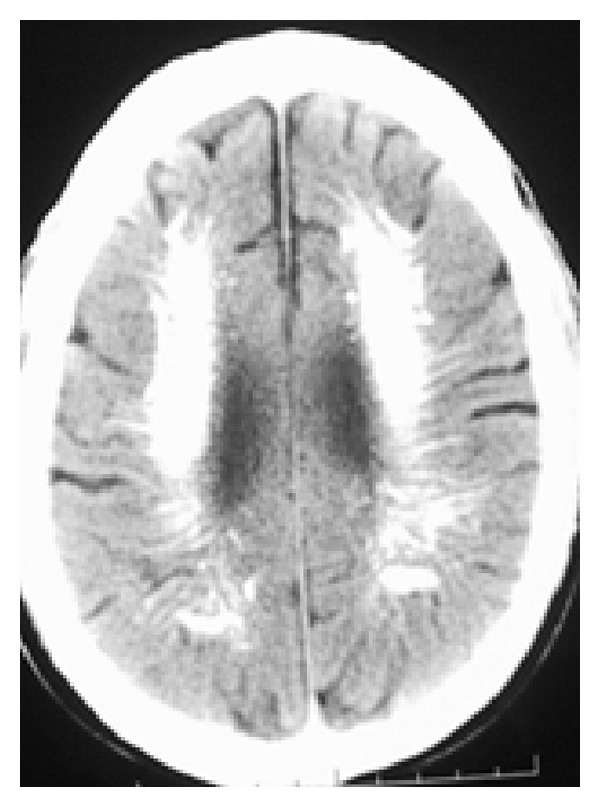
CT scan axial view demonstrating extensive bilateral calcifications in the periventricular white matter, semiovale center, and corona radiate.

**Figure 3:**
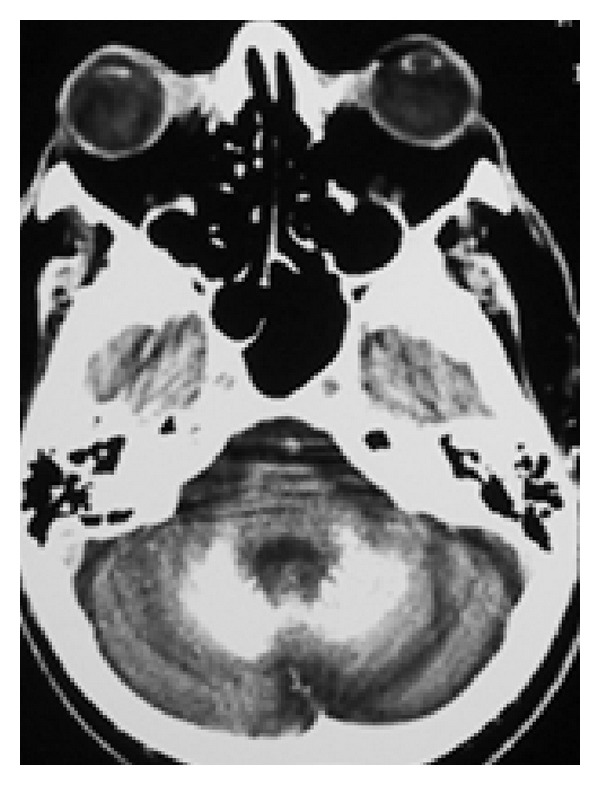
CT scan axial view demonstrating calcifications in both cerebellar hemispheres.

A 69-year-old man presented to the outpatient clinic 48 hours after an episode of loss of consciousness lasting a few seconds. He had a medical history of hypertension and hyperlipidaemia well controlled with medicine and a history of total thyroidectomy 18 years ago treated since then with levothyroxine. The patient's clinical course after surgery is unknown, as he rejected any postoperative followup, and the effort made to obtain any past medical records was ineffective. Nevertheless, the patient did report exophthalmos, excessive weight loss, and a large goitre before surgery, as well as calcium supplementation for a month after surgery, which he stopped taking by himself. Following the aforementioned patient's statements, Grave's disease was presumed to be the indication for total thyroidectomy. Neurological examination showed only mild extrapyramidal signs (mild rigidity and bradykinesia but not rest tremor) and the presence of more than one primitive reflex. A brain computed tomography (CT) scan revealed extensive bilateral symmetrical brain calcifications in the frontal lobes, basal ganglia, subcortical and periventricular white matter, and in the cerebellar hemispheres (Figures 1, 2, and 3). Minimental scale examination revealed a score of 24/30 (noted that the patient had only primary education). Family members reported no profound signs of dementia or incapability throughout patient's everyday life. The patient himself reported fatigability and a tingling sensation around the mouth. On investigation, his calcium level was 5.3 mg/dL (normal values (nv) 8.5–10.5 mg/dL), serum albumin 4.0 g/dL (nv 3.5–5 g/dL), serum magnesium 2.22 mg/dL (nv 1.58–2.55 mg/dL), serum phosphate 5.4 mg/dL (nv 2.5–5 mg/dL), PTH was undetectable, and TSH was normal 2.2 *μ*U/mL (nv 0.27–4.2 *μ*U/mL). The rest of the laboratory evaluation was unremarkable. An EEG showed evolution of alpha dominance and rare slow wave bursts of theta and delta activity. The clinical presentation of our patient was attributed to hypoparathyroidism. To prevent further complications from hypoparathyroidism, patient was prescribed with calcitriol 0.5 *μ*g/day and calcium 1 gm/day. With normalization of serum calcium levels (8.7 mg/dL), patient had improvement of extrapyramidal signs and complete diminish of paresthesias. Patient was followed up for a year, and he remained in good mental and physical health.

## 3. Discussion

Postoperative hypoparathyroidism is the most common complication of complete or near-complete extirpation of thyroid gland, by destruction or vascular compromise of parathyroid tissue [2]. Several thyroid conditions such as Grave's disease, thyrotoxicosis as a result of hyperactive thyroid adenomas, recurrent goiter, and thyroid carcinoma carry a higher risk to develop transient and permanent hypoparathyroidism postoperatively [3]. The main clinical features of hypoparathyroidism are a result of induced hypocalcaemia and can range from a life threatening condition to an asymptomatic laboratory finding [4]. Hypocalcaemia most commonly presents with paresthesia, cramps, muscle spasms, circumoral numbness, and seizures but can also present with laryngospasm, neuromuscular irritability, cognitive impairment, personality disturbances, prolonged QT intervals, electrocardiographic changes that mimic myocardial infarction, or heart failure [5].

Intracranial calcification is one of the features of chronic hypocalcaemia, and the calcifications typically involve the basal ganglia, thalami, and the cerebellum [6]. In our patient, calcinosis exceeds the common brain locations and involves the subcortical white matter of the frontal and parietal lobes. A review of the literature reveals only few case reports of excessive calcification of subcortical white matter regarding postoperative hypoparathyroidism [7–10]. The most commonly reported manifestations of postoperative hypoparathyroidism with basal ganglia calcification are parkinsonism [11] and seizures [12–14]. There are also reports of cognitive impairment [15] and even intracerebral hemorrhage [16]. The remarkable point of our case is the discordance between imaging and clinical symptoms and signs. Despite the wide brain calcification, the patient had only mild symptoms and signs.

The pathogenic mechanism of brain calcinosis in postoperative hypoparathyroidism is not yet defined. Although Virchow [17] and Bamberger and Von Rokitansky [18] independently described the histology of bilateral basal ganglia calcifications in 1855, it was not until 1939 that their association with chronic hypoparathyroidism was recognized by Eaton et al. [19]. Microscopic colloid deposition around cerebral blood vessels is followed by calcification most commonly in the basal ganglia [20]. According to Goswami et al. the progression of basal ganglia calcification is related to the calcium/phosphorus ratio [21]; thus, a strict control of hypocalcaemia and hypophosphatemia upon diagnosis is mandatory.

## 4. Conclusion

Chronic hypocalcaemia due to postoperative hypoparathyroidism can remain subclinical for long and detection of intracranial calcifications can be the trigger for further investigation with measurement of calcium and phosphorus levels. Calcium supplementation protects the patient from further complications of chronic hypoparathyroidism.
